# Clinical, Demographic, and Criminal Behavior Characteristics of Patients With Intellectual Disabilities in a Canadian Forensic Program

**DOI:** 10.3389/fpsyt.2019.00760

**Published:** 2019-10-15

**Authors:** Ipsita Ray, Alexander I. F. Simpson, Roland M. Jones, Kristina Shatokhina, Anupam Thakur, Benoit H. Mulsant

**Affiliations:** ^1^Complex Care and Recovery Program, Centre for Addiction and Mental Health, Toronto, ON, Canada; ^2^Department of Psychiatry, University of Toronto, Toronto, ON, Canada

**Keywords:** intellectual disability, forensic mental health, behavioral incidents, risk assessment, offending behaviour

## Abstract

**Background:** People with intellectual disability (ID) and forensic issues constitute a challenging clinical group that has been understudied in forensic settings.

**Methods:** We assessed the characteristics of patients with ID under the authority of the Ontario Review Board (ORB) in a large forensic program of a tertiary psychiatric hospital (excluding those with a cognitive disorder) and compared their characteristics with those of a non-ID control group.

**Results:** Among 510 adult ORB patients, 47 had an ID diagnosis. ID patients were of younger age at index offense, with a lower level of education, and were less likely to have been married or employed, more likely to have committed a sexual offense, more likely to have a diagnosis of paraphilia, less likely to be “not criminally responsible,” and more likely to be “unfit to stand trial.” They were also more likely to have committed their index offenses against care professionals and be treated in a secure unit.

**Conclusion:** Our findings have major implications for clinicians, clinical leaders, and policymakers about the specific needs of patients with ID presenting with forensic issues and differing needs in terms of treatment and risk management.

## Introduction

People with concurrent intellectual disability (ID) and forensic issues constitute a challenging clinical group in inpatient psychiatric settings and in the community. A few studies have characterized the offending behaviors, clinical characteristics, and outcomes in this group (1–8) (**Table 1**). The majority of forensic ID research is from the United Kingdom, where specialized ID forensic mental health services are well established, including forensic inpatient settings, community samples, and juvenile populations (15, 18). In these varying settings, the prevalence of reported ID has varied widely. In prison studies (as opposed to secure hospital studies), the prevalence of ID was typically reported to be around 1% to 2% and always below 5% (2, 3, 12).

**Table 1:** Summary of published studies of intellectual disability in forensic patients.

Author, Year of publication	Setting (location)	Sample size/study design/ID prevalence	Key findings
Alexander et al. (9)	Secure Intellectual Disability services in the UK	N = 362; ID-PD group, N = 48; ID group, N = 97; PD group, N = 217 Retrospective case-note analysis of patients discharged from four independent sector medium secure facilities.	Gender difference noted across groups. Over 30% females in ID-PD group and PD group. ID group, only 11% were females. PD group had a higher number of previous convictions, with almost 50% having over 10 convictions, compared to only a quarter in the other two groups. The PD group was significantly younger at first conviction than each of the other two groups. Previous admissions were less in the ID group than in the ID-PD group.
Barron et al. (10)	Specialist health and social services for people with ID; non-specialist services in the criminal justice system or (forensic) mental health system (UK)	N = 61 (53 males and 8 females with ID) Observational study assessing factors that influence outcome in ID offenders.	ID offenders started offending at an early age and had a history of multiple offenses. The most common index offense was violence with high rates of sex offending or arson. Half of the sample reoffended after a mean follow-up of 10 months.
Billstedt et al. (2)	Prison (Sweden)	N = 270 (sentenced male offenders 18–25 years age) Observational study ID: 1%	
Chester et al. (11)	High Secure hospital (England)	ID = 66/401 (16.5%) File review was done and questionnaires completed. Between-group analysis for patients with and without ID were compared	ID group had significantly shorter length of stay, fewer criminal sections, restriction orders, and prison transfers. ID group had higher levels of behavioral incidents and risk assessment scores.
Fazel et al. (12)	Prisons (10 surveys from 4 different countries: Australia, Dubai, New Zealand and USA)	N = 11,969 prisoners (92% males; 8% females) Review of reported prevalence of ID in general prison populations Prevalence: see box on the right	Most survey reported a prevalence of ID between 0.5% and 1.5%. Pooled prevalence from two screening studies: 6.1% (325/5329 prisoners). Overall pooled prevalence rate not estimated due to heterogeneity in studies.
Fitzgerald et al. (13)	Four medium secure units in the UK	ID-N = 25, control group-N = 45 Physical aggression measured over six months. HCR-20 and VRAG were done.	Both the instruments (HCR-20 and VRAG) consistently produced large effect sizes. They were able to predict any physical aggression.
Glaser and Florio (8)	Community forensic dual disability clinic (Australia)	N = 24; 23 males, 1 female Observational study assessing of characteristics of patients admitted to a community forensic dual disability clinic during the first 10 months of its operation	Offenders with ID were males and older in age and had continuing serious behavioral disturbances independent of their psychiatric diagnoses. One-third had a diagnosable major nonparaphilic psychiatric disorder; two-thirds had chronic medical illness.
Hassiotis et al. (14)	Prisons (UK)	N = 3563 Retrospective analysis of prison survey data ID: 170/3563 (4%); 126 males and 44 females	Prisoners with ID were more likely to be young females, had higher rates of self-harm and attempted suicide, were more likely to be on remand, had shorter sentence and lower reported social support.
Haysom et al. (15)	Juvenile custodial centres (Australia)	N = 295 (87% males; mean age: 17 years) Observational study 135/295 (46%) had borderline or low intellectual functioning	In Aboriginal participants, incarceration from a younger age significantly correlated with possible ID. Those with psychosis were 20% more likely to have possible ID compared to those without psychosis.
Lindsay et al. (10)	Forensic ID Service (Scotland)	N = 309 (males = 282, females = 27) 22-year (1986–2008) follow-up of cohort of offenders referred to specialist forensic ID services	Rate of mental illness was high, and higher in women (70%). Recidivism rates were high: 43% of general male offender group reoffended.
Lunsky et al. (6)	Inpatients on forensic and non-forensic units at 9 tertiary mental health hospitals (Canada)	N = 2218 inpatients Comparative study across three groups: Non-ID patients on forensic units = 506 ID on forensic units = 74 (60 males; 14 females) ID on nonforensic units = 282	Forensic inpatients with ID were more likely to be males, have a diagnosis of personality disorder, and have problems with arson. They were less likely to have a diagnosis of a mood disorder, substance misuse, or psychotic disorder
McCarthy et al. (3)	Male prison (UK)	N = 240 Observational study comparing characteristics of NDD and non-NDD groups 33/240 (14%) of subjects had ID	Offenders with NDD were younger and more likely to be homeless or unemployed; those with ID not reported separately from persons with other NDDs.
Nixon et al. (16)	Disability services (Australia)	N = 2220 (people with ID) 1310 males, 910 females Data-linkage study comparing ID and non-ID in community sample	People with ID were at higher risk of criminal charges and victimization for violent and sexual crimes.
O’Shea et al. (17)	Secure mental health settings (UK)	ID N = 109; non-ID N = 504 A pseudoprospective cohort study of the predictive efficacy of the historical, clinical and risk management - 20 (HCR -20) for those with ID.	The Historical, Clinical and Risk Management -20 (HCR -20) score significantly predicted physical aggression in both the groups. The ID group had higher scores on the historical and risk management items.
Raina and Lunsky (7)	Psychiatric hospital (Canada)	N = 39 forensic inpatients with ID compared with 39 nonforensic inpatients with ID Retrospective chart review	Forensic inpatients with ID were more likely to have a diagnosis of psychotic disorder and to have a history of drug and alcohol use
Stahlberg et al. (18)	Adolescents consecutively committed to juvenile institutions (Sweden)	N = 100 Observational study Prevalence: see box on the right	73% of participants with at least one major DSM-IV disorder diagnosis (excluding conduct disorder and substance abuse) including 11/100 (11%) with ID
Vicenzutto et al. (1)	Secure Psychiatric Hospital (Belgium)	N = 290 stabilized patients Observational study comparing people with Low IQ, low IQ and mental health illness (low IQ MHI), forensic inpatients with normal IQ and with or without mental illness (control group)	22.7% of participants presented with low IQ and mental health illness in the forensic population sample; the group represented a prevalence of 55.2% in forensic patients with low IQ and low IQ MHI. Higher proportion of mental illness in low IQ MHI group as compared to control group. Low IQ group had significantly more sexual offenses compared to Low IQ MHI and control group. Low IQ MHI group had significantly more nonsexual violent offenses and nonviolent nonsexual offenses compares with low IQ group.

ID, intellectual disability; NDD, neurodevelopmental disorders.

In both prison and hospital studies, those with ID were typically younger than those without ID (3, 6, 14), and their most common offenses were violent or sexual offenses or arson (6, 19). Those with ID had higher rates of self-harm and attempted suicide (4), higher rates of aggression (2), and a higher likelihood of comorbid personality disorder and substance use (6, 19, 20). People with ID and comorbid personality disorders had higher risk assessment scores compared to those with personality disorders in the secure hospital setting (9). This can be related to factors intrinsic to ID such as communication difficulties and impulse control. Very few of these studies of persons with ID in prison or forensic settings have characterized them comprehensively along all these various dimensions. Thus, we assessed the sociodemographic, clinical, and forensic characteristics of patients in a large hospital-based forensic service. We also assessed differences between patients with ID and a non-ID control group. Based on the literature, we hypothesized that the ID group would present with more aggressive behaviors and conflicts with peers and staff than the non-ID group and, as a result, would be more likely to become involved in episodes of physical restraints and locked seclusion.

## Materials and Methods

### Setting

The Centre for Addiction and Mental Health (CAMH) is a 530-bed psychiatric hospital located in Toronto, Ontario, Canada. It includes a forensic program with 190 inpatient beds and approximately 300 community forensic patients serving the Greater Toronto Area with a population of approximately 2.4 million people living in the catchment area. All forensic patients found to be unfit to stand trial (UST) or not criminally responsible (NCR) are under the jurisdiction of the Ontario Review Board (ORB). They undergo a formal review at least on an annual basis, and a comprehensive report is provided to the ORB before it hears evidence and gives its written disposition with reasons. Starting in the 1970s, reports prepared for annual ORB hearings of CAMH inpatients and outpatients and the disposition following the hearings have been collected in a registry. Typically, these forensic reports consist of 6 to 20 pages of text summarizing the patient’s clinical, diagnostic, personal, and legal history. For each report in the registry, a trained research analyst extracted and entered 101 variables into a database (e.g., gender, age, marital status, number of times in locked seclusion) organized into 10 sections (e.g., Demographics, Personal and Developmental History, Course in Hospital/Community While on Disposition Order). For the current study, the completion of analyses using this forensic case registry was approved by the CAMH Research Ethics Board before the start of this analysis.

### Participants

For this study, we selected the most recent ORB report for all cases coded and entered in the database between April 20, 2012, and August 28, 2017, after excluding 35 patients with a diagnosis of cognitive disorder or dementia. The resulting sample comprises 510 forensic patients who came under the jurisdiction of the ORB between September 18, 1990, and May 12, 2012. At the time of their most recent ORB report, all were 18 years or older; 191 were inpatients on a unit of CAMH Forensic Program, 18 were inpatients on another unit of CAMH, 293 were outpatients, two had absconded, and the current setting was not indicated for six.

### Measures

We extracted data from the patient’s most recent ORB report using methods previously described (21, 22). In summary, two senior researchers in the forensic program at the hospital where data were collected developed a coding scheme that contained all relevant demographic, clinical, legal, and risk variables of interest, as well as their operational definitions (protocol available through corresponding author). Variables were coded from psychiatric reports and ORB Reasons for Disposition reports. These reports typically contain comprehensive information about patients, including psychosocial history (e.g., childhood, relationships, education, employment history), criminal history (usually obtained from police records), mental health (e.g., diagnoses, hospitalizations), and risk assessments. Observations made during previous and current hospitalizations are recorded in these reports. Two research analysts (one master’s level; one with a bachelor’s degree) were trained on how to use the coding form and the Historical, Clinical and Risk Management -20 (HCR-20). During the training phase, there was a good interrater reliability between each research analyst and each of the two senior researchers for all variables (intraclass correlation coefficient, single measure [ICC1] >0.75; κ coefficient for categorical variables >0.75). Following the establishment of rater reliability, research analysts coded cases independently, and interrater reliability during data collection was again assessed by having the senior researchers code 20% of all cases coded by the research analysts. Again, ICC1 and κ values were always greater than 0.75, as suggested by Fleiss (23).

*Diagnoses* for participants were extracted from the clinical files and were recorded by the attending psychiatrist responsible for the patient’s care and made according to *Diagnostic and Statistical Manual of Mental Disorders, Fourth Edition* criteria (24). All clinical diagnoses that had been recorded by the attending psychiatrist were extracted for further analysis.

*Legal status* of the participants falls under the auspices of the ORB, which has two categories, namely, NCR or UST.

*The index offense(s)* includes the offense(s) for which there is an NCR or unfit finding.

*Charges* refer to the recorded offense for which the individual was found criminally responsible or UST.

*Victim* in the index offense(s) was defined as anyone who was considered to be directly harmed (physically or psychologically) by the patient during the offense.

*Victim’s relationship with the participant* was categorized as being *family* (spouse, parent, sibling, children, extended family), *copatient* or *roommate*, or *care professionals* (anyone who works with the patient within the institutional/healthcare setting). A *stranger* was defined as a victim who was completely unknown to the person.

*Locked seclusion* in a clinical setting is when a patient is placed in a room designed for this purpose with doors locked to manage risk of harm either to themselves or others. Coding for locked seclusion was based on at least one event occurring during the past year.

*Restraints* are either pharmacological or mechanical. Coding for restraints was based on at least one such event occurring during the past year.

*Conflict with staff or copatients* was coded in the registry as present when there was “general argumentativeness” or “not getting along with others.”

*Difficult behavior* was codes as “difficult to manage from the point of view of staff.” Examples include unruly behavior and noncompliance with unit rules.

*Victimization* was coded as “being bullied, picked on, insulted, verbally or physically assaulted.”

*Suicidal ideation* refers to presence of plans or thoughts of killing oneself.

*Homicidal/violent ideation* refers to plans, ideas, thoughts, desires, or urges about killing or harming someone.

### Data Analysis

We used descriptive statistics (frequencies, means, standard deviations, and percentages) to summarize sociodemographic, clinical, and forensic characteristics of ID and non-ID groups. χ^2^ Analyses were used to compare ID and non-ID groups for categorical variables, and Fisher exact tests were used where cell sizes for expected values were five or less. We calculated Mantel–Haenszel odds ratios (ORs) and 95% confidence intervals (CIs) as an effect measure. Pearson *r* was used for comparing continuous variables. All analyses were conducted using Stata 14.2 (25).

## Results

### Sociodemographic Characteristics and Clinical Comorbidity

The dataset included 510 participants, 47 of whom (9.2%) had a diagnosis of ID. Four had diagnoses of both ID and autism spectrum disorder (ASD). Three had a diagnosis of autistic spectrum disorder only and were excluded from the analyses as they formed a small group clinically distinct from both the ID and the non-ID groups. **Table 2** compares the sociodemographic and clinical comorbidity between the ID and non-ID groups. The majority of participants were males in both groups. Compared to the non-ID participants, ID participants were less likely to have completed at least 12 years of education (OR, 0.47; 95% CI, 0.29–0.77), less likely be married (OR, 0.26; 95% CI, 0.11–0.59) or employed (OR, 0; 95% CI, 0–0), and more likely to have a paraphilic disorder (OR, 5.35; 95% CI, 1.72–16.58).

**Table 2:** Sociodemographic and clinical characteristics of patients with and without an intellectual disability.

	With ID n (%)	Without ID n (%)	χ^2^, df	OR	95% CI	*p*
**Gender (N = 507)**						
Males	41 (87.2)	378 (82.2)	0.76, 1	0.67	0.28–1.64	0.38
Females	6 (12.8)	82 (17.8)				
**Education (N = 488)**						
Up to grade 8	5 (11.6)	31 (7.0)	Fisher exact	0.47	0.28–0.77	<0.01
Grades 8–11	27 (62.8)	188 (42.3)				
Grade 12 and above	11 (25.6)	226 (50.8)				
**Marital status at the time of index offense (N = 493)**						
Married/common law	7 (15.2)	184 (41.2)	Fisher exact	0.26	0.11–0.59	<0.001
Unmarried	39 (84.8)	263 (58.8)				
**Employment status at the time of index offense (N = 477)**						
Employed	0 (0.0)	45 (10.5)	Fisher exact	0	0–0	0.02
Unemployed	47 (100.0)	385 (89.5)				
Psychiatric diagnoses other than ID*						
Psychosis and related disorders	39 (83.0)	419 (91.1)	3.21, 1	0.48	0.21–1.09	0.07
Mood disorders	0 (0.0)	11 (2.4)	Fisher exact	0	0–0	0.61
Anxiety disorders	0 (0.0)	4 (0.9)	Fisher exact	0	0–0	1.00
Personality disorders	16 (34.0)	101 (22.0)	3.51, 1	1.83	0.96–3.50	0.06
Substance use disorders	23 (48.9)	241 (52.4)	0.20, 1	0.87	0.48–1.59	0.65
Paraphilias	5 (10.6)	10 (2.2)	10.61, 1	5.35	1.72–16.58	< 0.01

*ID, intellectual disability.

Missing data for marital status from non-ID group, n = 12. Missing data for employed from non-ID group, n = 30. Missing data for education from ID group, n = 5 and non-ID group, n = 15. Missing data for marital status from ID group, n = 0 and non-ID group, n = 12.

### Forensic Trajectory and Characteristics

**Table 3** summarizes the forensic characteristics of participants: compared to non-ID participants, ID participants were younger at the time of committing their index offense by about 4 years (*P* = 0.03); they were more likely to have committed a sexual offense (OR, 3.21; 95% CI, 1.46–7.03); 95% CI, the victims of the index offense were more likely to be professionals involved in their care (6.21; 95% CI, 2.54–15.17); and they were more likely to have been found UST and less likely to have been found NCR (OR, 3.73; 95% CI, 1.73–8.04) and more likely to be in a secure unit (OR, 0.74; 95% CI, 0.58–0.93).

**Table 3:** Forensic characteristics of patients with and without intellectual disability.

	With ID	Without ID	*t*	*p*		
	Mean (SD)	Mean (SD)				
Age at first admission	26.9 (17.5)	31.3 (17.7)	1.62	0.11		
Age at first arrest	28.5 (18.4)	31.3 (18.6)	0.99	0.32		
Age at index offense	31.8 (11.8)	35.6 (11.4)	2.18	0.03		
Age at the time of ORB report	38.1 (10.2)	41.4 (13.6)	1.61	0.11		
	**n (%)**	**n (%)**	**χ** **^2^**	**OR**	**95% CI**	***p***
**Legal status**						
Not criminally responsible	36 (76.6)	427 (92.4)	13.00	3.73	1.73-8.04	<0.001
Unfit to stand trial	11 (23.4)	35 (7.6)				
**Location at time of report**						
Secure	17 (36.2)	90 (20.6)	6.92	0.74	0.58-0.93	0.03
General	9 (19.2)	75 (17.2)				
Outpatient	21 (44.7)	272 (62.2)				
**Relationship with victim**						
Family (spouse, parent, sibling, children, extended family)	10 (21.3)	116 (25.1)	0.33	0.81	0.39-1.68	0.57
Copatient/cotenant/neighbor/roommate	7 (14.9)	67 (14.5)	0.01	1.03	0.44-2.41	0.94
Stranger	17 (36.2)	182 (39.3)	0.18	0.87	0.47-1.63	0.67
Care professional	9 (19.2)	17 (3.7)	21.13	6.21	2.54-15.17	<0.001
Friend	0 (0)	12 (2.6)	Fisher exact	0	0-0	0.61
Officer	6 (12.8)	56 (12.1)	0.02	1.06	0.43-2.62	0.89
Colleague	0 (0)	6 (1.3)	Fisher exact	0	0-0	1.00
No victim	6 (12.8)	42 (9.1)	0.68	1.47	0.59-3.66	0.41
**Type of index offense**						
Violence (assault, murder, attempted murder, weapons charges)	24(70.6)	259 (70.3)	0.00	1.01	0.47-2.20	0.97
Sexual offenses	10 (21.3)	36 (7.8)	9.48	3.21	1.46-7.03	< 0.01
Theft/break and enter/robbery	3 (6.4)	33 (7.1)	Fisher exact	0.89	0.26-3.02	1.00
Failure to comply	18 (38.3)	108 (23.3)	5.14	2.04	1.09-3.83	0.02
Utter threats	12 (25.5)	84 (18.1)	1.52	1.55	0.77-3.11	0.22
Abduction	1 (2.1)	14 (3.0)	Fisher exact	0.70	0.09-5.44	1.00
Mischief	3 (6.4)	55 (11.9)	Fisher exact	0.51	0.15-1.69	0.34
Arson	1 (2.1)	24 (5.2)	Fisher exact	0.40	0.05-3.02	0.72
Criminal harassment	2 (4.3)	36 (7.8)	Fisher exact	0.53	0.12-2.27	0.56

*ID, intellectual disability; ORB, Ontario Review Board.*

Missing data for Sexual offenses in ID group, n = 1.

### Behavioral Incidents

**Table 4** describes behavioral incidents among inpatient participants. There was little difference in most behavioral variables or clinical outcomes across most areas, including no difference in need for restrictive care practices.

**Table 4:** Past-year behavioral incidents of inpatients with and without intellectual disability.

	With ID n (%)	Without ID n (%)	X^2^	OR	95% CI	*p*
Locked seclusion^1^	7 (26.9)	45 (25.4)	0.03	1.08	0.43–2.75	0.87
Restraint	0 (0)	10 (5.6)	Fisher exact	0	0-0	0.37
Physical assault	12 (44.4)	55 (30.9)	1.96	1.79	0.78–4.10	0.16
Verbal assault^2^	17 (63.0)	79 (44.9)	3.07	2.09	0.90–4.86	0.08
Suicide attempt	1 (3.7)	2 (1.1)	Fisher exact	3.38	0.29–39.14	0.37
Homicide attempt	0 (0)	1 (0.6)	Fisher exact	0	0–0	1.00
Suicidal ideation	1 (3.7)	9 (5.1)	Fisher exact	3.36	0.94–11.97	0.07
Self-injurious behavior	3 (11.1)	10 (5.6)	Fisher exact	2.10	0.54–8.24	0.39
Homicidal/violent ideation	0 (0)	24 (13.5)	Fisher exact	0.53	0.26–1.08	0.07
Conflict with staff	16 (59.3)	86 (48.3)	1.12	1.56	0.68–3.56	0.29
Conflict with copatients	16 (59.3)	73 (41.0)	3.18	2.09	0.91–4.81	0.08
Overall difficult behavior	21 (77.8)	123 (69.1)	0.84	1.57	0.60–4.11	0.36
Being victimized	5 (18.5)	25 (14.0)	0.38	1.39	0.48–4.03	0.54
Absconding incidents	4 (14.8)	29 (16.3)	Fisher exact	0.89	0.29–2.78	1.00
New charges under the ORB^3^	0 (0)	6 (3.4)	Fisher exact	0	0–0	1.00

*ID, intellectual disability; ORB, Ontario Review Board.*

^1^Missing data for locked seclusion in ID group, n = 2.

^2^Missing data for verbal assault in ID group, n = 1 and non-ID group, n = 1.

^3^Missing data for new charges under the ORB in non-ID group, n = 1.

## Discussion

We assessed the characteristics of forensic patients with ID in one large Canadian hospital-based forensic mental health program and compared them with the characteristics of forensic patients without ID. In addition to clinical variables and inpatient behavioral incidents, this study provides a comprehensive analysis of offense characteristics of ID patients.

Approximately 10% of forensic patients in this program had a diagnosis of ID. Compared to those without ID, ID patients were younger, with a lower level of education, less likely to have been married or employed, less likely to be NCR, more likely to have committed a sexual offense, more likely to have a diagnosis of paraphilia, and more likely to be UST. Contrary to our hypotheses, we did not find that the ID group presented with more aggressive behaviors and conflicts with peers or that they were more likely to become involved in episodes of physical restraint or locked seclusion than those without ID. We, however, did find that those with ID were more likely to be detained in a higher level of security than those without ID. These findings and their implications deserve some comments.

Units with higher levels of security are associated with higher staff-to-patient ratios and higher levels of physical and procedural security, and therefore these factors may reduce and deter the likelihood of behavioral incidents resulting on those units. The 10% of patients with ID among forensic patients we found is similar to the proportion of 13% reported in a previous Canadian study (6) but different from the proportions reported in some other studies (**Table 1**). However, most of these other studies were carried out in different settings including some in prisons (2, 3, 12) or specialized settings for patients with ID (4, 9, 13, 26). This is unlike the present study, which was conducted on a forensic mental health population representative of one geographical area. The high prevalence of ID in male forensic patients seen in this study has been observed in other studies (6, 8) and is congruent with the higher prevalence of males in the general forensic population. By contrast, Anckarsäter et al. (27) reported a higher prevalence of ASD in females.

The underidentification of intellectual disabilities and ASD in secure settings including forensic psychiatric units has been emphasized in previous studies (12), and indeed, there may be missed cases in our series also. Prevalence estimates can also differ due to differences in definitions, diagnostic methods, and study methodology used. Better detection of neurodevelopmental disorders in forensic settings can influence prevalence estimates. Conversely, among people diagnosed with intellectual disabilities, a proportion of criminal offending may go undetected or underreported. People with more severe ID are less likely to enter forensic services and are more likely to be served in ID services (10).

As in some other studies (3, 13), we found that, on average, ID forensic patients committed their index offense at a younger age. Our participants with ID tended to be younger, on average, at the time of their index offense. Early intervention for this group could help reduce the risk of offending. As expected, ID patients had completed fewer years of formal education, similar to other reports (3). This emphasizes the societal challenge and potential benefits of providing appropriate educational experiences to people with ID. ID patients in our sample were much more likely to be UST and less likely to be found NCR than those without ID. This has implications for patients who are unfit due to their ID as opposed to those who are unfit due to a psychotic illness in that the former may not be amenable to change.

While literature about specific kinds of offending in this population is scant, those in high security settings had higher rates of comorbid personality disorder, complex presentations, and fatal or nonfatal interpersonal violence (20). In the same study, conviction for at least one sexual offense was present in more than 50% of the ID cases. In our sample, sexual offenses by ID patients were significantly more frequent than by non-ID patients. Notably, a higher rate of sexual recidivism in offenders with ID has been reported in follow-up studies (28). Some of the possible reasons for higher rates of sexual offending compared to those without include a higher incidence of sexual naivety, a lack of ability to form normal sexual relationships, and difficulties with impulse control (28). Whether this finding is related to a lack of developmental sociosexual knowledge needs further exploration.

We saw a higher rate of paraphilias in our ID participants, which is congruent with the association among paraphilias and lower levels of intellectual or adaptive functioning reported previously (1, 16, 29, 30). This higher rate of paraphilic behaviors has been attributed to lower levels of social awareness and behavioral self-control (31). Similarly, patients with ID had higher rates of both offenses involving professional caregivers and conflicts with copatients. The findings endorse higher levels of incidents reported in other studies (13, 17, 32, 33). Although participants with ID in a forensic setting constitute a minority group, their high level of needs, especially behavioral difficulties, may require a different approach. People with ID need more intensive support, which may not be adequately provided in general forensic services (6, 11).

These findings have important service implications. Patients with ID in the forensic mental health system may need different approaches to treatment, clinical support, and risk management The literature supports that individuals with ID often have communication difficulties, which usually increase with the severity of ID (34). Therefore, assessments of ID patients should routinely include a psychological assessment for cognitive and adaptive functioning. Typically, general forensic clinical settings do not cater to special needs, such as communication and learning difficulties, which can be seen in ID forensic patients. Specific training for staff on forensic psychiatry units in managing behaviors of ID patients could help these staff members mitigate violence-related risks and better support the needs of ID patients.

### Strengths of the Study

This study examined the diagnosis of ID in a complete population of all patients in a large forensic hospital using a comprehensive number of measures. Notably, there are very few studies outside Europe that have studied this population. The findings have significant implications for clinicians, clinical leaders, and policymakers about needs of this specific subgroup of patients presenting with forensic issues.

### Limitations

There are a number of limitations of this study. First, the past-year behavioral incidents recorded in **Table 4** were collapsed into binary variables, which recorded whether there was at least one occurrence of the behavior of interest. There is likely to have been a wide variation in frequencies within these variables, but we were unable to investigate any relationship between ID and severity of the behavioral problem. Our approach was necessary as precise counts of the occurrence of each variable were not available to us, but further studies to investigate frequency and severity of behavioral problems are recommended. Second, although our data extraction methods have been validated, there is a possibility that incidents may be underreported, particularly less serious incidents, as they may not have been included in the narrative reports from which we extracted data. This underreporting is probably as likely in the ID and non-ID groups and therefore unlikely to introduce bias when carrying out group comparisons. However, overall, we may have underestimated the proportion of people having engaged in these disruptive behaviors. Third, in our analyses, we have carried out multiple group comparisons, which increases the risk of obtaining type I errors. One approach would be to have carried out Bonferroni adjustments; however, the risk of type II errors is inflated, and the approach is not widely recommended (35). Although the risk of type I errors remains, we have instead reported ORs with CIs to aid interpretation of the findings. Fourth, the study involves data collected from the forensic division of one Canadian psychiatric hospital. Therefore, the findings may not generalize to other hospitals or settings. Fifth, standardized screening tools were not used on the entire population, so significant comorbidities such as attention-deficit/hyperactivity disorder were not consistently sought. Lastly, as this was a cross-sectional study, we could not assess and compare longitudinal outcomes in the two groups.

## Summary and Future Directions

The findings from this study suggest that there is a small but significant number of patients with ID assessed and treated in the forensic mental health system. These vulnerable patients present with some important differences in characteristics and needs. The lack of specialized services for people with ID has been associated with an increased likelihood of future offending (3). Together, these findings support recommendations for the development of specialized forensic units for managing patients with ID. Additional longitudinal studies examining both clinical outcomes and costs are needed to further inform policy decisions.

Future studies also need to explore the severity as well as the frequencies of behavioral incidents occurring, relationship among risk factors, length of hospital stay, subsequent community integration, and risk of reoffending. Given the relatively small number of forensic patients with ID, the creation of regional or national database could facilitate our understanding of the needs of these patients.

## Ethics Statement

For the current study, the gathering of data in the Forensic Case Register and the completion of analyses using this forensic case registry was approved by the CAMH Research Ethics Board.

## Author Contributions

IR, BM, AS, and AT: conception and design of the project, interpretation of data, and manuscript drafting. RJ: conception and design of the project, analysis and interpretation of data, and manuscript drafting. KS: data entry, analysis, and interpretation.

## Conflict of Interest

The authors declare that the research was conducted in the absence of any commercial or financial relationships that could be construed as a potential conflict of interest.
